# Antitumor efficacy of combination of interferon-gamma-inducible protein 10 gene with gemcitabine, a study in murine model

**DOI:** 10.1186/1756-9966-27-63

**Published:** 2008-11-05

**Authors:** Kai Mei, Lian Wang, Ling Tian, Jingrui Yu, Zhixuan Zhang, Yuquan Wei

**Affiliations:** 1 Department of Oncology, Sichuan Cancer Hospital, Chengdu, 610041, PR China; 2 State Key Laboratory of Biotherapy and Cancer Center, West China Hospital, West China Medical School, Sichuan University, Chengdu, 610041, PR China; 3 Department of Pathology, West China College of Stomatology, Sichuan University, Chengdu, 610041, PR China

## Abstract

**Background:**

Interferon-γ-inducible protein 10 (IP-10) is a potent inhibitor of tumor angiogenesis. It has been reported that the antiangiogenic therapy combined with chemotherapy has synergistic effects.

**Methods:**

To elucidate the mechanisms of IP-10 gene combined with a chemotherapy agent, we intramuscularly injected pBLAST-IP-10 expression plasmid combined with gemcitabine into tumor-bearing mice.

**Results:**

The proliferation of endothelial cells was effectively inhibited by IP-10 combined with gemcitabine *in vitro*. Treatment with pBLAST-IP-10 twice a week for 4 weeks combined with gemcitabine 10 mg/kg (once a week) resulted in sustained high level of IP-10 protein in serum, inhibition of tumor growth and prolongation of the survival of tumor-bearing mice. Compared with administration of IP-10 plasmid or gemcitabine alone, the angiogenesis in tumors were apparently inhibited, and the numbers of apoptotic cells and lymphocytes in tumor increased in the combination therapy group.

**Conclusion:**

Our data indicate that the gene therapy of antiangiogenesis by intramuscular delivery of plasmid DNA encoding IP-10 combined with gemcitabine has synergistic effects on tomor by inhibiting the proliferation of endothelail cells, inducing the apoptosis of tumor cells, and recruiting lymphocytes to tumor in murine models. The present findings provided evidence of antitumor effects of genetherapy combined with chemotherapy.

## Background

Angiogenesis, the formation of new blood vessels, is important not only for normal physiological processes, but also for the development of pathologic conditions such as cancer, rheumatoid and inflammation. Presently, accumulated evidences indicate that the growth and metastasis of solid tumors is dependent on angiogenesis. Therefore, targeting tumor vasculature has been a popular strategy of therapeutics [1,2]. But anti-angiogenesis alone is not sufficient due to the angiogenesis-independent phase of tumor growth. It has been reported that cytotoxic agents can impair neovasculature directly or indirectly [3]. Further, proliferative endothelial cells in new vessels are sensitive to cytotoxic agents [4]. Combination therapy, consisting of low-dose chemotherapy and antiangiogenesis, may produce improved efficacy and reduced toxicity due to the synergistic effect on tumors [4,5].

Interferon-γ-inducible protein 10 (IP-10), a member of the α chemokine family, is secreted by activated T cells, fibroblast and endothelial cells [6]. It attracts activated, but not resting, T lymphocytes and NK cells [7-9] via stimulating CXCR3 chemokine receptor [7]. Tumor cell lines stably transfected with the IP-10 gene were rejected by immune-system [10]. Intratumoral injection of IP-10 gene facilitated regression of established mice tumors [11]. IP-10 is also a potent inhibitor of angiogenesis [10,12]. Thus, we hypothesize that IP-10, which is involved in immune (T and NK cells) and antiangiogenic responses, is a good candidate for treatment of malignant tumors.

Gemcitabine (GEM), a deoxycytidine analog, is currently used as a therapeutic agent against several solid tumors, such as non-small cell lung cancer (NSCLC), pancreatic cancer and bladder cancer [13]. In local advanced or metastatic NSCLC, gemcitabine has been used as the first-line therapeutic. However, the efficacy of gemcitabine is unsatisfactory with a response rate of only about 36%, and time of tumor progression is 4 to 5 months. [14] Therefore we sought to develop therapeutic agents that would increase the anti-tumor efficacy of gemcitabine. IP-10 may enhance the effects of gemcitabine in combination therapy via angiogenic-independent mechanisms. In 2005, a synergistic effect of their combination on solid tumors was found by our study group [15]. In this study, we try to elucidate the mechanism of combination of IP-10 with gemcitabine.

## Materials and methods

### Preparation of IP-10 plasmid

pBLAST-IP-10 plasmid (purchased from Invivogen, San Diego, CA, USA) was transformed into *E*. coli JM109, and cultured at 37°C for plasmid isolation by endotoxin-free plasmid maxiprep kit (Roche, Germany). The concentration of pBLAST-IP-10 plasmid was determined by ultraviolet spectrophotometer.

### Transfection of COS cells with pBLAST-IP-10

COS cells were plated in six-well plates at 2 × 10^5 ^cells/well in DMEM containing 10% FBS at 37°C overnight. Cells at 60–80% confluence were transfected with 2 μg of plasmid and 6 μl lipofectamine (Invitrogen) in serum-free DMEM following the manufacture's instructions. After addition of the lipofectamine-DNA complex, the cells were incubated at 37°C for 12 h. Then the complex was replaced with normal growth medium and the cells were incubated at 37°C for an additional 60 h before collection of the conditioned medium.

### Western blot analysis

IP-10 protein produced by pBLAST transfected COS cell was analyzed by Western blot. Briefly, ten microliters of the conditioned media were mixed with 2 × sodium dodecyl sulfate sample buffer and were separated on a 12% SDS-PAGE gel. Gels were electroblotted with Sartoblot on to a poly (vinylidene difluoride) membrane. The membrane blots were blocked in 5% non-fat dry milk, washed, and probed with rabbit-anti-murine IP-10 antibody (Pepro Tech House, USA) at 0.2 μg/ml at 4°C overnight. Blots were then washed and incubated with a biotinylated secondary antibody (biotinylated goat anti-rabbit IgG), followed by transfer to Vectastain ABC (Vector Laboratories, Burlingame, CA, USA) [16].

### Effect of IP-10 and gemcitabine on endothelial cell *in vitro*

Biological activity of IP-10 and gemcitabine on HUVEC was determined using conditioned medium from transfected cells in endothelial cell proliferation assay. Briefly, 3 × 10^3 ^endothelial cells (human umbilical vein endothelial cells, HUVECs; and a cell line of mouse endothelial cells, SVECs) and non-endothelial cells (NIH/3T3 fibroblast) were plated on to 96-well culture plates in triplicate, and incubated (37°C, 5% CO_2_) for 24 h in 100 μl medium containing bFGF. Then the medium was replaced with 40 μl of normal saline or IP-10-containing supernatant from transfected COS cells or control cells. Gemcitabine was added at final concentration from 20 μM to 100 μM. After incubation for 20 min, 60 μl of medium was added. After another 72 h of incubation, the cell number was determined by trypan blue dye exclusion test, and the percent growth inhibition of HUVECs and NIH/3T3 fibroblast was calculated by the following formula: inhibition % = [(N-N_T_)/(N-N_0_)] × 100, where N is the number of cells in the untreated control cultures after n days, N_0 _is the cell number on day 0, and N_T _is the number of cells in the treatment group after n days [16,17].

### Tumor models

Tumor cell injections were carried out using freshly prepared cell suspensions at a concentration of 1 × 10^7 ^cells/ml in PBS. C57BL/6 and BALB/c female mice about 20 g body weight were inoculated subcutaneously (s. c.) in the right flank with 1 × 10^6 ^LL/2c Lewis lung cancer cells and H22 hepatocarcinoma cells, respectively, as previously described [18].

### Tumor growth inhibition study

After tumor model had been established, the mice were weighed, coded and randomly divided into different groups with 10 mice per group. Mice were injected with different doses of IP-10 plasmid (100 μg, 50 μg, 12.5 μg) into both quadriceps twice weekly for 4 weeks (empty plasmid and saline as controls). At the conclusion of the treatment, tumor volume was measured. The optimal dose of IP-10 plasmid was selected for use in combination therapy with gemcitabine. Gemcitabine (Lilly Pharmaceutical Co. U.S.A) was administered alone or in combination with IP-10 plasmid by i.p. injections (10 mg/kg, 1/12 of the maximal tolerance dose) on days 14 and 21 after initial of IP-10 plasmid injection. Additional control animals were injected with normal saline 100 μl. The doses of gemcitabine selected were determined after optimum dose experiment (data not shown). Survival times and tumor volumes were observed.

### ELISA determination of IP-10 expression

The levels of IP-10 in serum after intramuscular administration of IP-10 plasmid were assayed by ELISA. In brief, 96-well microtiter plates (Falcon) were coated with rabbit-anti-IP-10 antibody in 100 mM coating buffer (carbonate-bicarbonate, pH 9.6) at 4°C overnight and blocked with 150 μl/well of 1% bovine serum albumin in phosphate-buffered saline (PBS) at 37°C for 1 h. Sera diluted 1/5 (100 μl/well) was added and incubated for 2 h at 37°C. Horseradish peroxidase-conjugated goat anti-rabbit IgG (Sigma, St Louis, MO, USA) diluted 1/5000 in PBS-Tween-BSA buffer was added (100 μl/well) and incubated for 1 h at 37°C. After washing four times with PBST, ABTS (100 μl/well) (Sigma) was added for 30 min, and absorbance was measured at 450 nm with an ELISA reader (BioRad, Hercules, CA, USA). A dilution series of recombinant IP-10 was used as a standard. Assays were done in triplicate.

### Alginate encapsulation assay

Alginate-encapsulated tumor cell assays were performed as described previously [19]. Meth A cells were directly dispersed in a 1.5% solution of sodium alginate after centrifugation and added dropwise into a swirling solution of 250 mM calcium chloride at 37°C. Each alginate bead contained approximately 1 × 10^5 ^tumor cells. Mice were then anesthetized, 4 beads were implanted subcutaneously into an incision made on the dorsal side, and then the incisions were sutured. Mice were randomized into four groups (10 mice per group) and treated with normal saline, IP-10 plasmid alone, gemcitabine alone or IP-10 plasmid combined with gemcitabine. Treatment was initiated on days 2 following beads implantation with injection of IP-10 plasmid i.m. (100 μg/day for each mouse, twice weekly over a 2-week period) in both quadriceps, or gemcitabine i. p. twice (10 mg/kg) on days 5 and 11, or both agents together. Normal saline (100 μl) was administered as control. Twelve days after implanting alginate beads, the mice were injected intravenously with 100 μl of a 100 mg/kg fluorescein isothiocyanate (FITC)-dextran solution (Sigma). Alginate beads were surgically removed, and FITC-dextran was quantified against a standard curve of FITC-dextran.

### Histological analysis

To assess the formation of blood vessel of tumor tissues, immunohistochemistry was done as described [20]. Frozen sections were fixed in acetone and incubated with a monoclonal rabbit anti-mouse CD31 at a 1:200 dilution (Pharmingen, San Diego, CA, USA) at 4°C overnight. After sections were washed with PBS, biotinylated rabbit anti-mouse secondary antibody was added at a 1:100 dilution (Dako, Carpinteria, CA, USA). The sections were then stained with labeled streptavidin biotin reagents (Dako LSAB kit, peroxidase). Vessel density was determined by counting the number of microvessels per high-power field in the sections [17,21].

Apoptotic cells were identified by TUNEL assay (an *in situ *apoptotic cell detection kit based on the enzymatic addition of deoxyadenine-nucleotide to the nicked DNA by terminal deoxynucleotidyl transferase) following the protocol supplied by manufacture (Boehringer Mannheim).

Tumor tissues from each group were fixed in 10% neutral buffered formalin solution, embedded in paraffin. Sections 3–5 μm thick were stained with H&E according to standard procedure [22]. Pathologists were blinded for quantitation of infiltrating lymphocytes as previously described [23].

To identify possible side effects resulting from IP-10 plasmid and gemcitabine, sections of paraffin-embedded tissues, including liver, kidney, lung, heart and spleen, were stained by H&E.

### Statistic analysis

Data is expressed as mean ± SD. Statistical significance was determined by Student's t test. Kaplan-Meier curves were established for the survival time of mice from each group and survival was compared by means of the log-rank test. Differences between variables or ranks were considered significant when yielding a P < 0.05.

## Results

### Expression of biologically active IP-10 *in vitro*

**Figure 1:**
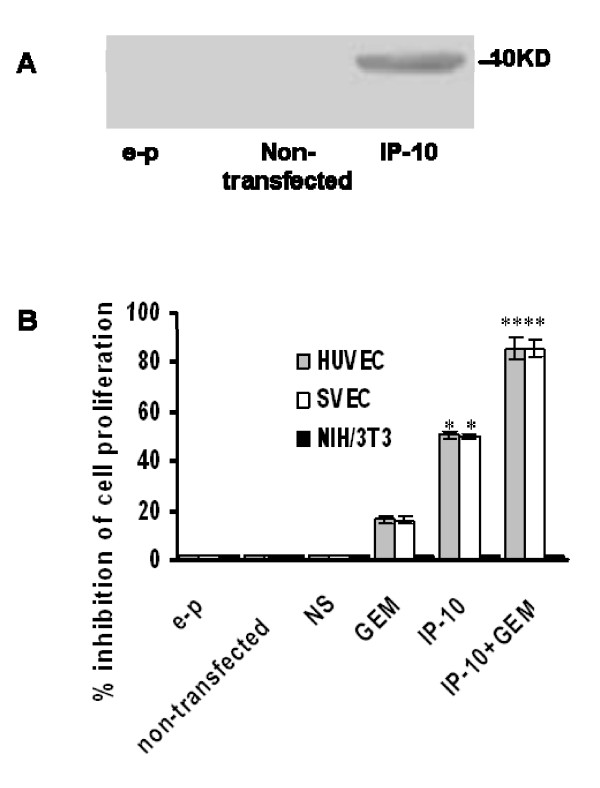
**Expression of IP-10 *in vitro *after transfection into COS cells**. IP-10 expression *in vitro *and inhibition proliferation of endothelial cells by IP-10 combined with gemcitabine. A. Western blotting analysis of secreted IP-10 by COS cells. Conditioned media was obtained from COS cells transfected with empty plasmid or pBLAST-IP-10, or from non-transfected cells. The protein mass molecular markers are indicated on the right. B. Inhibition of endothelial cell proliferation by IP-10 combined with gemcitabine. Exponentially growing HUVEC, SVEC, NIH/3T3 were exposed to the conditioned medium from COS cells transfected with pBLAST-IP-10 for 72 h, or exposed to normal saline and gemcitabine. The rate of cell proliferation inhibition was calculated. Combination treatment significantly inhibited the proliferation of endothelial cells compared with gemcitabine alone, or IP-10 alone or other controls. *, *P *< 0.05; and **, *P *< 0.01.

Large-scale endotoxin-free expression pBLAST-IP-10 plasmid was purified and transfected into COS cells. Western blot analysis was used to determine expression of IP-10 protein (Fig. 1A). Conditioned medium was obtained from COS cells transfected with empty plasmid (lane a), non-transfected cells (lane b) and cells transfected with pBLAST-IP-10 (lane c). IP-10 immunoreactivity at ~10 KD was only identified in the lane from the transfected cultures.

### Inhibition of endothelial cell proliferation by IP-10 combined with gemcitabine

The biological activity of secreted IP-10 was tested against human umbilical vein endothelial cells (HUVECs primary cells) and a cell line of mouse endothelial cells (SVEC). As a control, the conditioned medium was also tested against non-endothelial cells (mouse NIH/3T3 fibroblast cells). Compared with the medium from the COS cells transfected with control vector or nontransfected cells, conditioned medium from IP-10-transfected COS cells inhibited HUVEC and SVEC endothelial cell growth. However, it had no effect on proliferation of non-endothelial cells (Fig. 1B). The proliferation of endothelial cells was inhibited by IP-10 combined with gemcitabine, compared with normal saline, IP-10 alone or gemcitabine alone.

### Serum level of IP-10 after intramuscular administration of plasmid pBLAST-IP-10

**Figure 2:**
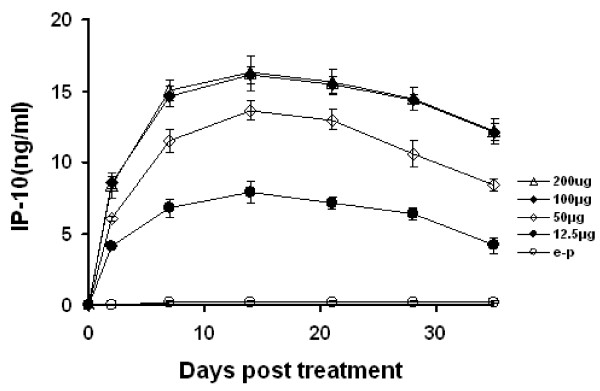
**Increased serum levels of IP-10 after intramuscular administration of IP-10 plasmid**. Empty plasmid (control, 100 μg, open circles) or selected doses of IP-10 plasmid (12.5 μg, solid circles, 50 μg, open rhombus, 100 μg, solid rhombus, 200 μg, open triangles) were administered by intramuscular injection. Mice were treated twice weekly over a 4-week period. Serum was collected on days 0, 2, 7, 14, 21, 28 and 35. IP-10 protein was determined by ELISA.

Selected doses (12.5 μg, 50 μg, 100 μg and 200 μg) of pBLAST-IP-10 plasmid and empty plasmid were administered by intramuscular injection to mice twice a week for 4-week. Serum was collected on days 0, 2, 7, 14, 21, 28 and 35, and IP-10 levels were determined by ELISA. Treatment with pBLAST-IP-10 resulted in the elevated IP-10 serum levels compared with empty plasmid or normal saline (Fig. 2). In addition, similar levels of IP-10 protein were achieved by administration of 100 μg and 200 μg plasmid, and they were higher than those by administration of 12.5 μg and 50 μg plasmid, empty plasmid and normal saline.

### The antitumor effects of IP-10 plasmid combined with gemcitabine

**Figure 3:**
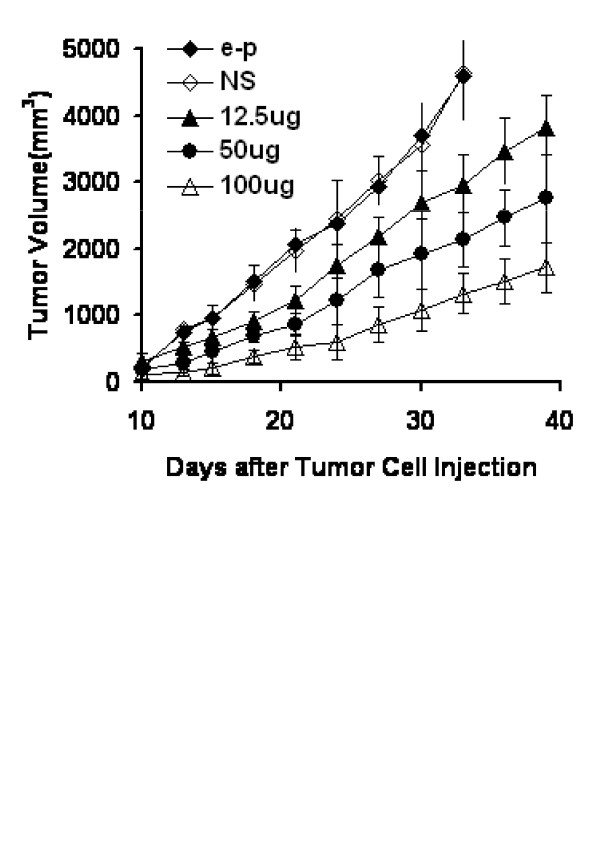
**Effects of DNA dose on tumor growth by intramuscular administration of pBLAST-IP-10**. Empty plasmid (control, 100 μg, open circles), normal saline (control, 100 μg, solid triangles) and selected doses of IP-10 plasmid (12.5 μg, solid circles, 50 μg, open rhombus, 100 μg, solid rhombus) were administered by intramuscular injection. Mice bearing Lewis lung cancer were injected twice weekly for 4 week.

Inhibition of tumor growth by IP-10 plasmid injection was found to be dose-dependent (Fig. 3). The mice were injected intramuscularly with IP-10 plasmid (12.5 μg, 50 μg and 100 μg), empty plasmid or normal saline. Injection with 100 μg of IP-10 plasmid twice weekly for 4 weeks induced reduced tumor volume compared with the controls.

**Figure 4:**
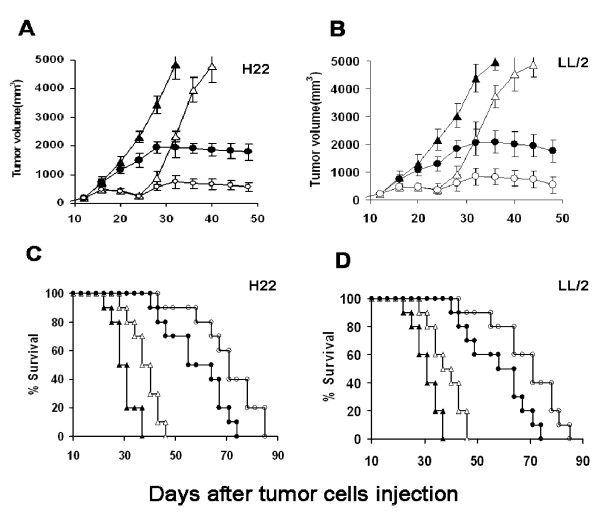
**Antitumor efficacy of IP-10 plasmid combined with gemcitabine in two murine models**. Mice (ten mice per group, including normal saline, solid triangles, gemcitabine alone, open triangles, IP-10 plasmid alone, solid circles, IP-10 plasmid combined with gemcitabine, open circles) were treated with injection of IP-10 plasmid 100 μg (i. m.) twice weekly for 4 weeks and/or administration of gemcitabine i.p. cycled twice (10 mg/kg, on the days 14 and 21 after initial of IP-10 plasmid administration). Normal saline 0.1 ml was injected as control. The treatment started at day 7 after 1 × 10^6 ^hepatocarcinoma cells (A, C) or Lewis lung cancer cells (B, D) were injected subcutaneously into mice. Combined treatment with IP-10 plasmid and gemcitabine resulted in significant inhibition of tumor growth and longer life span versus other three controls.

Treatment of the mice with combination therapy was initiated when tumors were palpable on 7 days following injection of H22 hepatocarcinoma or LL/2c Lewis lung cancer cells. In both tumor models, the combination therapy was consisted of intramuscular injection of pBLAST-IP-10 plasmid (100 μg) and intraperitoneal injection of gemcitabine (10 mg/kg). IP-10 plasmid was administered twice weekly for 4 weeks, and once-weekly administration of gemcitabine initiated a week following the first IP-10 injection. The combination therapy of IP-10 plasmid and gemcitabine resulted in inhibition of tumor growth (Fig. 4A–B). On the day 32, the tumor volume inhibition rates in combined therapy group were 85% and 81.2% in H22 and LL2 bearing mice respectively, which were higher than that in IP-10 therapy group (61.5% and 53% in H22 and LL2 respectively, both *P *< 0.01). Further, the prolonged survival of tumor-bearing mice was observed in the combination group, although both kinds of tumor-bearing mice died from progressive tumors. The average life spans of normal saline treated mice bearing with H22 and LL2 tumor were 30.7 days and 31 days respectively. They were increased to 37.9 days (H22) and 38.2 days (LL2) in gemcitabine group, and to 58.2 days (H22) and 57.6 days (LL2) in IP-10 group. In contrast, the combined treatment resulted in significant increases to 69 days in H22 bearing mice, and 70 days in LL2 bearing mice (*P *< 0.05, by log-rank test) (Figures 4C and 4D).

Gross measures of toxicity, such as alterations in weight loss, fur ruffling, life span, behavior and feeding, were not observed in any of the treatment groups. Further, microscopic examination of hematoxylin and eosin (H&E) stained tissue sections of heart, liver, spleen, lung, and kidney did not provide evidence of pathologic changes in any of the treatment groups.

### Inhibition of angiogenesis

**Figure 5:**
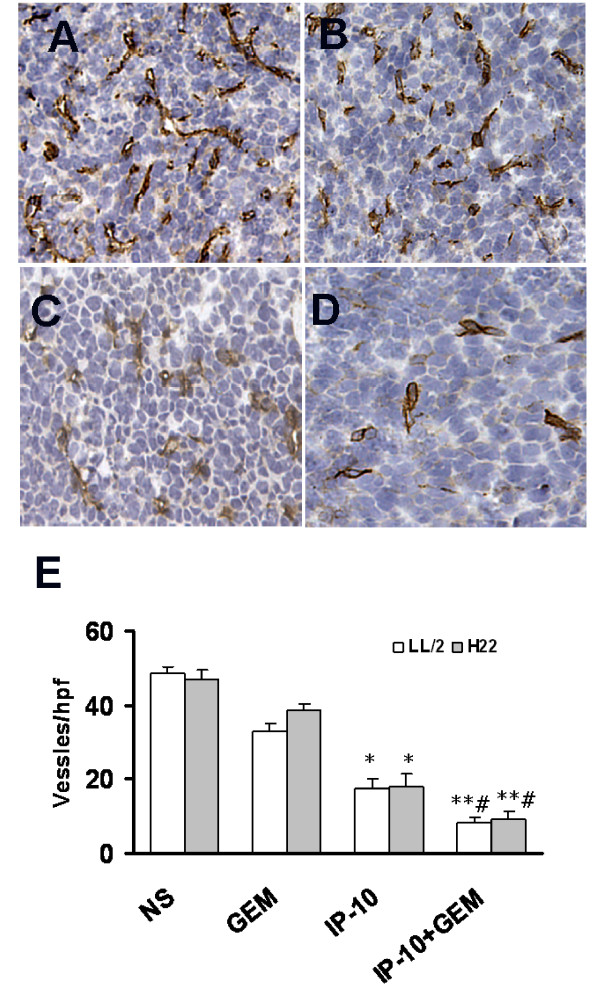
**Anti-angiogenesis assay by immunohistochemical analysis with CD31**. Inhibition of angiogenesis within tumor from IP-10 plasmid combined with gemcitabine therapy mice was estimated by immunohistochemical analysis. Sections of frozen LL/2 tumor tissues obtained from mice treated with normal saline (A), gemcitabine (B), IP-10 plasmid (C) and IP-10 plasmid combined with gemcitabine (D). Vessel density was determined by counting the number of microvessels per high-power field (E) in tumor sections stained with an antibody reactive to CD31 (brown). Original magnification, ×200 (A-D). *, *P *< 0.05, versus normal saline group; **, *P *< 0.01 versus gemcitabine and normal saline group; and ^#^, *P *< 0.05, versus IP-10 group.

The effects of IP-10 plasmid and gemcitabine on tumor vasculature were determined by immunohistochemical staining for CD31 and estimation of angiogenesis via microvessel counts. The combination of IP-10 plasmid and gemcitabine resulted in the inhibition of the angiogenesis in tumors (Fig. 5D) compared to controls (Fig. 5A–C). The numbers of vessels in tumor sections from mice in combined treatment group (8.51 ± 1.09/HPF and 9.13 ± 2.25/HPF in LL/2 and H22 respectively) were significantly less than that in IP-10 alone group (17.37 ± 2.75/HPF and 17.81 ± 3.85/HPF in LL/2 and H22 respectively, both *P *< 0.05) and other two control groups (*P *< 0.01, Fig. 5E).

**Figure 6:**
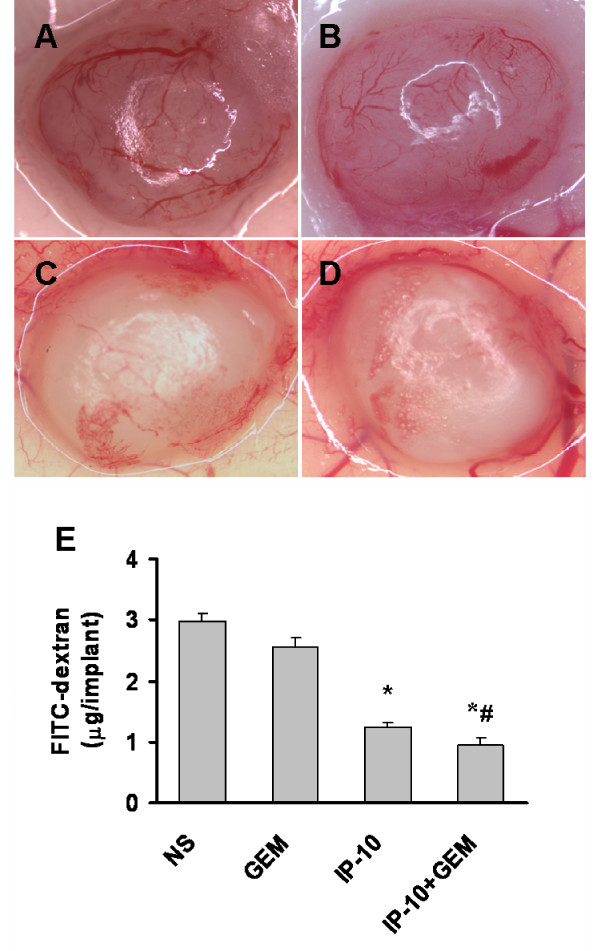
**Inhibition of angiogenesis assay by alginate beads *in vivo***. Beads containing 1 × 10^5 ^Meth A cells were implanted subcutaneously into BALB/c mice on day 7 after the treatment with IP-10 plasmid or gemcitabine. Twelve days later, beads were surgically removed, and FITC-dextran was quantified. FITC-dextran uptake (E) and representative photograph of alginate beads of normal saline (A), gemcitabine (B), IP-10 plasmid (C) and IP-10 plamsid combined with gemcitabine (D). The images of alginate implants and FITC-dextran uptake showed the reduction of vascularization in combination treatment and IP-10 plasmid treated alone group compared with normal saline controls. *, *P *< 0.01 versus normal saline and gemcitabine group; and ^#^, *P *< 0.01 versus IP-10 group.

The inhibition of angiogenesis in the combination therapy was confirmed by the alginate encapsulation assay. Angiogenesis was quantitated by measuring the uptake of FITC-dextran into implanted alginate beads. The vascularization of alginate beads was significantly reduced with 74% decreased FITC-dextran uptake in the combination therapy group (Fig. 6D) compared with normal saline group (Fig. 6A), while 57% decreased FITC-dextran uptake in IP-10 group (*P *< 0.05, Fig. 6C).

### Increased apoptosis in combination therapy group

**Figure 7:**
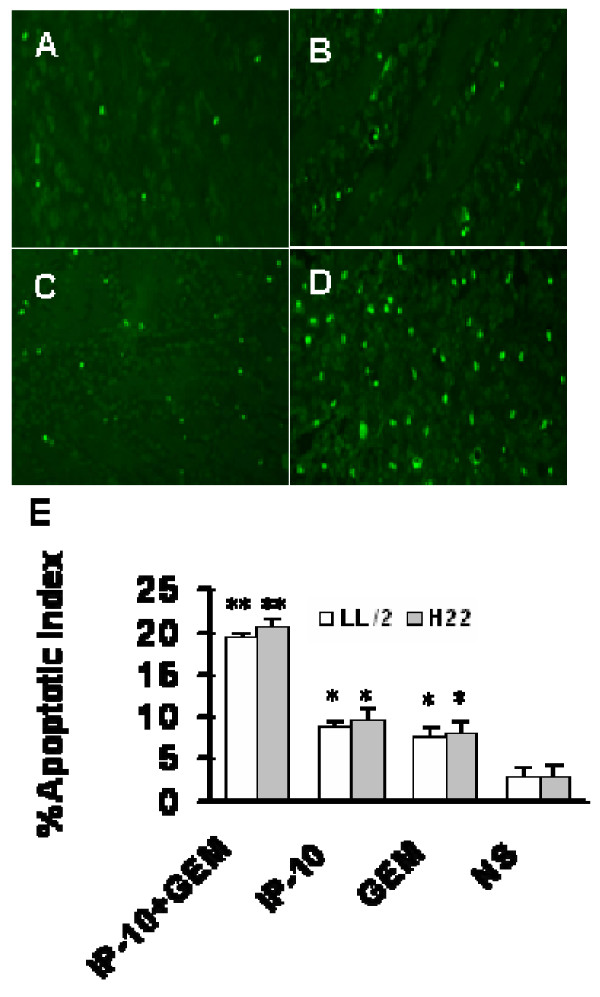
**Increased apoptosis in tumors after being treated with IP-10 plasmid**. Representative sections from LL/2 tumors were presented in normal saline (A), gemcitabine (B), IP-10 plasmid (C) and IP-10 plasmid combined with gemcitabine (D) groups. Bars represented apoptotic index of tumor tissues from LL/2 (open bars) and H22 (solid bars) (E). IP-10 plasmid combined with gemcitabine induced increased apoptosis compared with saline control (**, *P *< 0.01), also IP-10 plasmid alone or gemcitabine alone versus normal saline (*, *P *< 0.05).

To investigate the effect of combination of IP-10 plasmid DNA with gemcitabine on apoptosis of tumor cells, formaldehyde-fixed and paraffin embedded sections from tumor tissues were analyzed by TUNEL assay. Apoptotic cells with condensed chromatin dispersed throughout the sections. Treatment with IP-10 plasmid or gemcitabine alone slightly affected the apoptotic rate of tumor cells compared with normal saline (*P *< 0.05), whereas combined treatment induced increased number of apoptotic cells than other three controls (all *P *< 0.01, Fig. 7).

### Histological analysis

**Figure 8:**
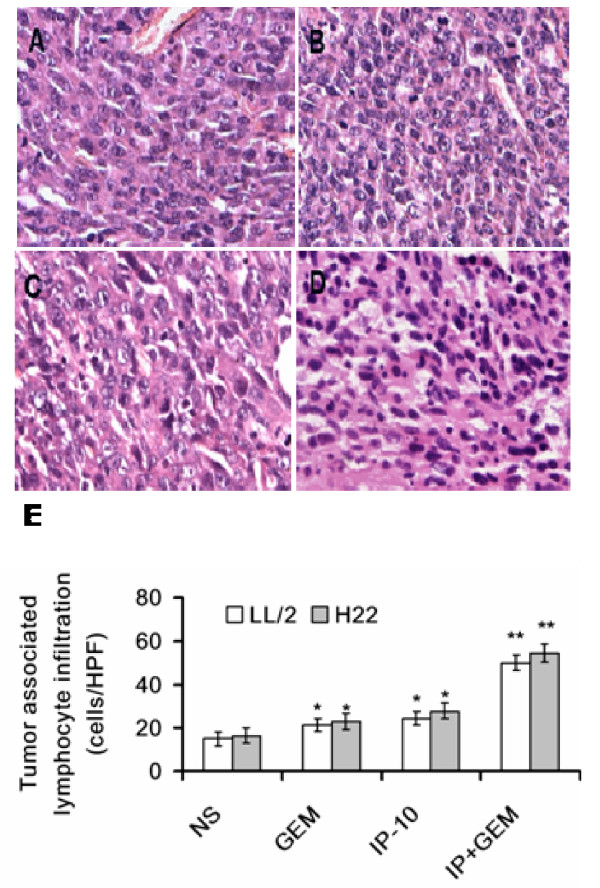
**Increased lymphocytes infiltration induced by IP-10 plasmid combined with GEM (× 200)**. Representative sections of four treated groups from H22 neoplasm tissue were photomicrographed at ×200 magnification (A-D). A few lymphocytes infiltrated in tumor tissues of normal saline group (A). Lymphocyte infiltration increased by administration of GEM (B) or IP-10 plasmid DNA (C). A further increase of lymphocyte infiltration was induced by IP-10 DNA combined with GEM (D) in H22 and LL/2 tumor tissues (E). *, *P *< 0.01 versus normal saline; and **, *P *< 0.01 versus GEM alone and IP-10 alone.

Combination treatment resulted in obvious lymphocyte infiltration in tumor tissues, compared with treatment of IP-10 plasmid, or gemcitabine alone, or normal saline. Representative sections of four treated groups from H22 neoplasm tissue were depicted (Fig. 8A–D). Quantitation of tumor-infiltrating lymphocytes revealed apparent differences in combination treated group relative to other three groups in both H22 and LL/2 neoplasm tissues (all *P *< 0.01, Fig. 8E).

Except for the mild degeneration of parenchyma cells, no obvious pathologic changes was found in H&E stained sections of heart, liver, spleen, lung and kidney.

## Discussion

Interferon-γ-inducible protein 10 (IP-10) is a potent inhibitor of angiogenesis, and may be a downstream mediator in interferon-induced antiangiogenesis. IP-10 plays an important role in antitumor activity via at least two possible mechanisms: 1) inhibition of tumor angiogenesis and 2) attraction of lymphocytes in the tumor tissue. It has been shown that intratumor administration of IP-10 plasmid induced tumor regression and inhibited tumor metastasis. IP-10 has also been investigated in combination with various cytokines against tumors [24]. In 2005, our study group developed a novel combination therapy consisting of IP-10 plasmid and a chemotherapeutic agent, gemcitabine (2', 2'-difluorodeoxycytidine), for the treatment of solid tumors [15].

Gemcitabine is a nucleoside analogue of cytidine, and its metabolites are embedded into DNA strand with the subsequent addition of one base, thus halting DNA synthesis. Cellular DNA repair systems are unable to correct for this process, leaving the cells vulnerable to apoptosis. Currently, gemcitabine is therapeutically used against a wide range of solid tumors as a single agent and in combination with other drugs, such as cisplatin [7,25]. In the present study, we found that combination IP-10 with gemcitabine resulted in prolonged survival and shrink tumors in a murine model, indicating its antitumor efficacy.

Chemotherapy agents have been reported to inhibit directly and indirectly the growth of vascular endothelial cells [3]. Gemcitabine inhibited the growth of HUVECs with an IC50 of 0.08 mM [26]. In our study, the proliferation of vascular endothelial cells was significantly inhibited *in vitro *by combination IP-10 with gemcitabine compared with IP-10 alone or gemcitabine alone, suggesting the antitumor mechanism of the combination therapy may be, at least in part, due to inhibition of the proliferation of vascular endothelial cells.

Gemcitabine is effective in inducing apoptosis of a variety of cancer cells, including non-small cell lung cancer, pancreatic, gastric and hepatic cancer [27]. IP-10 has been shown to inhibit tumor angiogenesis. We hypothesized that gemcitabine and IP-10 would act synergistically in their anti-angiogenesis and inducing apoptosis. In this study, decreased angiogenesis and increased apoptosis were observed when IP-10 was administered in combination with gemcitabine. The growth and proliferation of endothelial cells are stimulated, in part, by the vascular endothelial growth factor secreted spontaneously by tumor cells [28]. Increased apoptosis of tumor cells may result in reduced secretion of vascular endothelial growth factor from tumor cells, which may induce decreased angiogenesis. Intramuscular injection of IP-10 plasmid resulted in constitutively high expression levels *in vivo *(had been confirmed in our experiment). Vascular repair occurs during interphase necessitates the continuous administration of chemotherapy [29]. The possible inhibition of blood vessel repair induced by IP-10 explains the enhanced antitumor efficacy of the combination therapy in the current report.

It has been reported that the antitumor effect of IP-10 is dependent on T cells. Further, IP-10 has been shown to induce protective antitumor immunity via specific CD8^+ ^T cells [30]. IP-10 is also a chemoattractant for monocyte and T lymphocyte. Histologic analyses have demonstrated that lymphocytes and neutrophils infiltrated into the tumor region [8,31]. In our study, the combination treatment resulted in obvious lymphocyte infiltration in tumor tissues, compared to the IP-10 plasmid, GEM or normal saline treatments. The results suggest that lymphocyte infiltration may also account for the enhanced antitumor efficacy of combination treatment.

Chemotherapy agents often result in cytotoxic responses and are fraught with various side effects. Many of the pronounced side effects in conventional chemotherapy can be avoided with the application of low dose gemcitabine. In the present study, we selected low dose gemcitabine (10 mg/kg, 1/12 of maximum dose) in the combination therapy [13]. Based upon the resulting serum levels of IP-10, the 100 μg dose of plasmid per injection was identified as the optimal dose. In this study, no obvious side effects were observed in the combination treatment group, suggesting IP-10 and gemcitabine may be safely applied in combination protocols [32].

In conclusion, our study indicates that combination therapy with IP-10 plasmid and gemcitabine is a well-tolerated protocol with enhanced antitumor activity. This antitumor activity may be due, in part, to inhibition of angiogenesis via their effects on endothelial cells and attraction of lymphocytes into tumor tissues. These findings are important for further exploration of potential clinic application of the combined approach.

## Conclusion

In this study, we tested the mechanisms of a novel combination therapy consisting of IP-10 plasmid DNA and gemcitabine for the treatment of solid tumors. Gene therapy of anti-angiogenesis by intramuscular delivery of plasmid DNA encoding IP-10 combined with gemcitabine was effective in inhibition of angiogenesis by suppressing the proliferation of endothelial cells, and induction of lymphocytes infiltration in tumor tissues and the apoptosis of tumor cells in murine models. The present findings also provided evidence of antitumor effects of gene therapy combined with chemotherapy.

## Competing interests

The authors declare that they have no competing interests.

## Authors' contributions

KM and LW participated in its design, discussed the results, drafted the manuscript and carried out cell culture, western blot, ELISA and immunohistochemical and histological analysis. ZZ helped to establish animal tumor models. JY carried out experiments, data collection and statistical analysis, and helped to write the manuscript. YW and LT conceived of the study and participated in its design. All authors read and approved the final manuscript.
